# Comparison of the diagnostic power of serum circulating miR-21, VEGF, and CA15-3 in breast cancer

**DOI:** 10.3389/fonc.2026.1821704

**Published:** 2026-05-28

**Authors:** Hang Li, Qian Li, Xiaojing Tie, Feng Ji, LiNa Ma, Chunyan Ma

**Affiliations:** 1 Department of Laboratory Medicine, Kaifeng Central Hospital, Kaifeng, Henan, China; 2 Key Laboratory of Tumor Biomarker Detection of Kaifeng, Kaifeng, Henan, China; 3 Oncology Department, Kaifeng Central Hospital, Kaifeng, Henan, China; 4 Breast and Thyroid Department, Kaifeng Central Hospital, Kaifeng, Henan, China

**Keywords:** breast tumor, CA15-3, diagnostic value, serum circulating miR-21, VEGF

## Abstract

**Objective:**

The aim of this study was to assess the clinical utility of serum circulating microRNA-21 (miR-21) in the early diagnosis of breast cancer (BC), and to compare its diagnostic performance with raditional serum biomarkers, vascular endothelial growth factor (VEGF) and carbohydrate antigen 15-3 (CA15-3).

**Methods:**

From January 2023 to October 2025, a total of 106 women diagnosed with BC, 69 women with benign breast nodules, and 62 healthy women undergoing routine physical examinations were enrolled at a single institution. Serum levels of miR-21, VEGF, and CA15-3 were measured and compared across groups. Relationships between miR-21 and the traditional markers were analyzed using Spearman correlation analysis. Diagnostic efficacy was assessed via receiver operating characteristic (ROC) curve analysis.

**Results:**

Relative expression levels of serum miR-21, VEGF, and CA15-3 were significantly elevated in individuals with BC compared to healthy controls (all *p*<0.05). Serum miR-21 and CA15-3 levels were significantly higher in the BC group than in the benign nodule group (both *p*<0.05). No significant correlations were observed between miR-21 and VEGF (r = –0.032, *p*=0.747) or CA15-3 (r = –0.068, *p*=0.487) in the BC cohort. ROC curve analysis demonstrated that miR-21 had an area under the curve (AUC) of 0.897 for distinguishing BC from healthy individuals, with a sensitivity of 81.10% and a specificity of 98.40% at a cut-off value of 1.36. When differentiating BC from benign nodules, the AUC was 0.842, with a sensitivity of 76.40% and specificity of 98.60% at a cut-off value of 1.52. The diagnostic performance of miR-21 exceeded that of VEGF and CA15-3.

**Conclusion:**

Serum circulating miR-21 demonstrated significantly elevated expression in patients with BC and presented superior sensitivity and specificity compared to VEGF and CA15-3. These findings highlight miR-21 as a promising non-invasive biomarker for early breast cancer detection, particularly in distinguishing benign from malignant lesions, potentially reducing unnecessary biopsies. However, limitations include a relatively small sample size and single-center design, necessitating validation in larger, multicenter prospective cohorts. Future studies should explore miR-21’s association with molecular subtypes and clinical outcomes to facilitate clinical translation.

## 1 Introduction

Breast cancer (BC) remains a widespread disease worldwide, despite advances in its detection and treatment. According to the Analysis of Cancer Incidence and Mortality in China by Region, 2024 issued by the National Cancer Center in 2026, the incidence rate of female breast cancer in China is 54.54 per 100,000 population, making it the third most frequently diagnosed cancer in Chinese women after lung cancer and thyroid cancer. Moreover, the incidence rate shows a trend of being higher in urban areas than in rural areas (1). Early-stage disease often presents without specific clinical manifestations, and many patients receive a diagnosis at intermediate or advanced stages, thereby missing the optimal window for treatment. Early detection and timely intervention are associated with improved prognosis in patients with BC. Currently, laboratory markers commonly applied in clinical diagnosis, including carbohydrate antigen 15-3 (CA15-3) and carcinoembryonic antigen, serve primarily as tumor markers for monitoring disease progression in metastatic BC. However, these markers demonstrate limited sensitivity for localized lesions and reduced specificity, as elevated levels may occur in certain benign conditions and malignancies of other organs. Consequently, these markers are not suitable for BC screening or distinguish diagnosis (2, 3).

The vascular endothelial growth factor (VEGF) signaling pathway is a key regulatory axis indispensable for both physiological and pathological angiogenesis, vascular remodeling, and lymphangiogenesis. The growth, local invasion, and distant dissemination of solid tumors are entirely dependent on the nutritional and metabolic support provided by the tumor neovascular network. In the hypoxic tumor microenvironment, tumor cells and cancer-associated stromal cells are strongly induced to become overactivated, leading to sustained overexpression of VEGF and other pro-angiogenic mediators (4). Beyond its role in regulating vascular phenotypes, VEGF is also a central effector molecule that mediates the reprogramming of immunosuppressive tumor microenvironments. Previous mechanistic studies have confirmed that miR-21 acts as a critical upstream regulator of the VEGF pathway, directly driving its aberrant activation. This suggests that specific inhibition of the miR-21/VEGF signaling axis represents a promising strategy to reverse tumor immunosuppression and restore effective anti-tumor immune responses (5–7).

MicroRNAs (miRNAs) constitute a class of small non-coding RNAs involved in the regulation of gene expression and play important roles in tumor initiation and progression. MicroRNAs (miRNAs) are widely distributed in various extracellular body fluids, including serum, plasma, saliva, and urine. Circulating miRNAs remain highly stable by associating with vesicles or Argonaute proteins, enabling minimally invasive sampling via routine blood collection and efficient detection by reverse transcription quantitative polymerase chain reaction (RT-qPCR). Due to their unique stability and clinical accessibility, circulating miRNAs have emerged as promising biomarkers for early tumor screening, prognostic evaluation, and therapeutic target development. As a well-characterized oncogenic miRNA, miR-21 is frequently overexpressed in both tissue and circulation across multiple solid tumors, including breast cancer, and it regulates key malignant processes such as tumor cell proliferation, invasion, and metastasis (8, 9).

In the field of liquid biopsy and tumor biomarker research, RT-qPCR is a widely used and sensitive method for detecting specific miRNAs, including miR-21. Compared with other detection methods, Stem-Loop RT-qPCR allows efficient capture and precise quantification of trace amounts of miR-21 in the peripheral circulation (10). Using this platform, numerous studies have demonstrated that aberrant expression of circulating miR-21 is significantly associated with early detection, pathological progression, treatment response, and survival outcomes in patients with malignant tumors, highlighting its clinical value as a minimally invasive and repeatable liquid biopsy candidate marker (6, 11). In this study, we systematically evaluated the diagnostic performance of individual biomarkers using receiver operating characteristic (ROC) curve analysis, comparing their utility in distinguishing breast cancer from healthy individuals and benign breast lesions. Key indicators including the area under the curve (AUC), sensitivity, and specificity were analyzed. Specifically, we compared the diagnostic performance of serum circulating miR-21 with that of the clinically used biomarkers VEGF and CA15-3, objectively clarifying its advantages and limitations. We also explored the complementary potential of these markers when used in combination, providing a theoretical basis and supporting data for the development of multimodal liquid biopsy strategies in breast cancer.

## 2 Participants and methods

### 2.1 Research participants

A prospective cohort study design was adopted. Sample size estimation was conducted using PASS 15.0 software. According to the findings from a preliminary study (a difference in serum circulating miR-21 expression of 2.3 between the BC group and the healthy group, with a standard deviation of 0.75), the significance level was set at α = 0.05 (two-sided), and the statistical power was set at 1–β = 0.9.A minimum sample size of 82 individuals was calculated for the BC group. To account for potential hemolysis of serum samples and loss of detection data during the study (with an estimated dropout rate of approximately 29%), Therefore, an initial cohort of 130 cases was enrolled to ensure the final effective sample size. During the study, 24 non-compliant specimens were actually excluded, resulting in an actual dropout rate of 18.5%, with ultimately 106 valid cases included in the observation group (BC group). These patients ranged in age from 36 to 86 years, with a mean age of 55.12 ± 10.41 years.

Inclusion criteria were: a diagnosis of BC confirmed by histopathological examination in accordance with the diagnostic criteria outlined in the Guidelines for the Diagnosis and Treatment of Breast Cancer (2022 Edition) (2); newly diagnosed patients who had not received any anti-tumor treatment such as radiotherapy, chemotherapy, or surgery; and the availability of complete clinical data.

Exclusion criteria comprised: coexisting malignant tumors; severe cardiac, hepatic, or renal dysfunction; autoimmune or infectious diseases; and pregnancy or lactation. Detailed results are shown in **Table 1**.

**Table 1:** Inclusion and exclusion criteria for the three study groups.

Group	Inclusion criteria	Exclusion criteria
Healthy Control Group	1. Healthy females who underwent routine health examinations at our hospital during the same period. 2. No history of breast-related diseases, with no abnormal lesions/nodules on breast imaging.3. No acute or chronic infections or severe systemic underlying diseases in the past 3 months.4. Complete clinical data, voluntary participation, and signed informed consent.	1. History of any malignant tumor.2. Previous breast surgery, radiotherapy, chemotherapy, or endocrine therapy.3. Pregnant or lactating women.4. Missing clinical or imaging data.
Benign Breast Nodule Group	1. Confirmed benign breast nodules (e.g., fibroadenoma, proliferative nodules) by ultrasound, mammography, and postoperative/core needle biopsy pathology.2. Complete clinical, imaging, and pathological data.3. No prior anti-tumor intervention before surgery.4. Voluntary participation and signed informed consent.	1. Concurrent malignant breast lesions or other systemic malignancies.2. Previous breast-related treatment history.3. Severe hepatic or renal insufficiency, autoimmune diseases, or acute inflammatory conditions.4. Pregnant or lactating women.
Breast Cancer Group	1. Histopathologically confirmed primary breast cancer.2. No neoadjuvant radiotherapy, chemotherapy, endocrine therapy, or targeted therapy prior to surgery.3. Preoperative breast imaging completed with full documentation of nodule/lesion size parameters.4. Complete general and clinicopathological data.5. Voluntary participation and signed informed consent.	1. Breast cancer with distant metastasis or recurrent/metastatic disease.2. Concurrent primary malignant tumors of other organs.3. Severe systemic diseases such as severe infection or immunodeficiency.4. Missing key clinical data or failure to complete all index tests.

A total of 62 healthy participants who underwent physical examinations at the study center during the same period were enrolled as the healthy control group. These individuals, aged 34 to 86 years, with a mean age of 54.13 ± 9.76 years, had no history of breast disease or other malignancies. Laboratory results, including liver and kidney function tests and complete blood counts, were within normal ranges.

Additionally, 69 patients diagnosed with benign breast nodules were included as the benign nodule group during the same period. These patients ranged in age from 36 to 79 years, with a mean age of 54.07 ± 10.57 years, and included 38 cases of breast fibroadenoma, 23 cases of breast hyperplasia, and 8 cases of breast cysts. All diagnoses were confirmed by pathological or imaging examinations. None of the participants in this group had a history of malignancy.

No statistically significant differences in age were observed among the three groups (F=0.623, *p*=0.537), indicating comparability. This study was approved by the Medical Ethics Committee of the institution (ethics approval number: 2026ks-lw001).

### 2.2 Main reagents and instruments

A magnetic bead-based miRNA extraction kit (Tiangen Biotech, DP501), FastKing cDNA First-Strand Synthesis Kit II (Tiangen Biotech, KR116-02), and SuperReal PreMix Plus (Enhanced Version) for quantitative PCR (Tiangen Biotech, FP205-02) were utilized. VEGF levels were measured using a dry fluorescence immunoassay kit (Guangzhou Labsim Biotechnology Co., Ltd.), along with the matched AFS2000A instrument. CA15-3 levels were determined using an electrochemiluminescence immunoassay kit (Roche Diagnostics) and the corresponding E801 analyzer. Quantitative real-time PCR was performed on an ABI 7500 system (ABI, USA). Additional instruments used included a high-speed refrigerated centrifuge (Eppendorf, Germany, 5430R), a dry fluorescence immunoassay analyzer (Guangzhou Labsim Biotechnology Co., Ltd., AFS2000A), an electrochemiluminescence immunoassay analyzer (Roche Diagnostics, E801), and a constant temperature metal bath (Hangzhou Bioer Technology).

All reverse transcription primers and real-time quantitative PCR primers used in this study were independently designed by our research team. The primer sequences were subjected to specific alignment analysis using the NCBI Primer-BLAST tool. Post-reaction, amplification specificity was evaluated via melting curve analysis, which demonstrated single amplification peaks for all genes with no primer dimers or nonspecific products detected, ensuring the accuracy and reliability of subsequent experimental data. Specific primers for miR-21 were synthesized by Sangon Biotech (Shanghai) Co., Ltd., with the following sequences:

forward primer 5’-TAGCTTATCAGACTGATGTTGA-3’, reverse primer 5’-CAACATCAGTCTGATAAGCTA-3’; reference gene U6 snRNA primers: forward primer 5’-CTCGCTTCGGCAGCACA-3’, reverse primer 5’-AACGCTTCACGAATTTGCGT-3’. The reverse transcription primer 5’-GTCGTATCCAGTGCAGGGTCCGAGGTATTCGCACTGGATACGACTCAACA-3’.

### 2.3 Experimental methods

#### 2.3.1 Sample collection and processing

For all study participants, 5 mL of fasting venous blood was collected in the early morning and placed into vacuum tubes without anticoagulant. After standing at room temperature for 30 minutes, the samples were centrifuged at 3000 r/min for 10 minutes (centrifugal radius: 10cm) to separate the serum. The resulting serum was transferred into RNase-free centrifuge tubes, aliquoted, and stored at –80 °C until further analysis. Repeated freeze-thaw cycles were avoided. Strictly implement pre-analytical quality control: exclude specimens with hemolysis, lipemia, or jaundice, and discard non-compliant specimens; complete serum separation within 2 hours after blood collection to prevent RNA degradation due to prolonged storage; immediately transfer the separated serum into RNase-free centrifuge tubes and store at −80°C, avoiding repeated freeze-thaw cycles.

#### 2.3.2 Extraction of serum circulating miR-21

A 200 μL aliquot of serum was used for extraction, following the protocol provided with the magnetic bead-based miRNA extraction kit. First, 200 μL of lysis buffer RL containing β-mercaptoethanol (final concentration: 1%) was added. The mixture was vortexed for 30 seconds and allowed to stand at room temperature for 5 minutes. Subsequently, 200 μL of anhydrous ethanol was added, followed by vortexing and thorough mixing. The solution was transferred to an adsorption column CR3 and centrifuged at 12,000 r/min for 30 seconds; the waste was discarded.

Next, 500 μL of protein removal solution RW1 was added, followed by centrifugation at 12,000 r/min for 30 seconds. The waste was discarded. Then, 500 μL of wash solution RW (prepared with anhydrous ethanol) was added, followed by centrifugation at 12,000 r/min for 30 seconds; this wash step was repeated once. The adsorption column was transferred to a new collection tube and centrifuged at 12,000 r/min for 2 minutes to eliminate residual ethanol.

To elute the miRNA, 30 μL of RNase-free water was added to the center of the column membrane and incubated at room temperature for 2 minutes, followed by centrifugation at 12,000 r/min for 2 minutes. The eluate, containing the extracted miRNA, was collected and stored at –80 °C for later use.

#### 2.3.3 Reverse transcription reaction

A 20 μL reaction mixture was prepared in accordance with the instructions provided with the reverse transcription kit. Specifically, 2 μL of 5× gDNA Eraser Buffer, 1 μL of gDNA Eraser, 5 μL of miRNA template, and 2 μL of RNase-free water were combined. The mixture was incubated at 42 °C for 2 minutes to remove genomic DNA contamination and then cooled on ice. Subsequently, 2 μL of 10× FastKing RT Buffer, 1 μL of FastKing RT Enzyme Mix, 1 μL of miR-21–specific reverse transcription primer, and 6 μL of RNase-free water were added. The reaction was incubated at 42 °C for 30 minutes, followed by incubation at 95 °C for 5 minutes to inactivate the reverse transcriptase. The resulting cDNA was diluted tenfold with RNase-free water and stored at –20 °C for further use.

#### 2.3.4 Quantitative real-time PCR detection

A 20 μL reaction mixture was prepared in a 96-well PCR plate, consisting of 10 μL of 2× SuperReal Premix Plus, 0.8 μL of miR-21 forward primer (10 μmol/L), 0.8 μL of miR-21 reverse primer (10 μmol/L), 2 μL of cDNA template, 0.4 μL of 50× ROX Reference Dye, and 6 μL of RNase-free water. Each sample was run in triplicate. A no-template control and a positive control were included. Negative control reactions were performed using RNase-free water in place of the template to rule out contamination and non-specific amplification. Total RNA from human breast cancer MCF-7 cells served as the positive control to confirm the functionality of the reverse transcription and qPCR amplification systems.

The amplification protocol was as follows: pre-denaturation at 95 °C for 15 minutes; denaturation at 95 °C for 10 seconds; annealing at 60 °C for 30 seconds with synchronous fluorescence acquisition, for a total of 40 cycles. A melting curve analysis was performed under the following conditions: 95 °C for 15 seconds followed by 60 °C for 1 minute, and then 95 °C for 15 seconds, to verify the specificity of the amplification product.

The relative expression level of miR-21 was calculated using the 2^-ΔΔCt method, Briefly, with U6 snRNA serving as the internal reference gene. ΔCt was calculated as Ct(miR-21)-Ct(U6), and ΔΔCt was obtained by subtracting the mean ΔCt of the healthy control group from the ΔCt of each test sample.

#### 2.3.5 VEGF and CA15-3 testing

VEGF levels were measured using a dry fluorescence immunoassay, performed in accordance with the manufacturer’s instructions for the VEGF assay kit and AFS2000A instrument (Guangzhou Labsim Biotechnology Co., Ltd.). CA15-3 levels were determined via electrochemiluminescence immunoassay, following standardized procedures as outlined in the instructions for the Roche E801 instrument and the corresponding reagent kit. Serum VEGF and CA15-3 levels were measured using commercial assay kits according to the manufacturers’ instructions. The VEGF kit had a reference range of <160 ng/L and a linear range of 15-1400 ng/L, with intra-assay and inter-assay coefficients of variation (CV) ≤15%. The CA15-3 kit had a reference range of 0.00-26.40 U/mL and a linear range of 1.5–300 U/mL; samples exceeding the upper limit were diluted 1:10 to extend the reportable range to 3000 U/mL, with intermediate precision CV ≤20%. All measured values were within the linear range of the respective assays, and all procedures were performed under strict internal quality control.

### 2.4 Statistical methods

Data analysis and figure generation were performed using SPSS 26.0 statistical software and GraphPad Prism 9.0. The Shapiro–Wilk test was used to assess the normality of quantitative data. Data conforming to a normal distribution were expressed as mean ± standard deviation (x̄ ± s). Comparisons among multiple groups were conducted using one-way analysis of variance (ANOVA), and pairwise comparisons were carried out using the LSD-t test. Quantitative data not meeting normal distribution criteria were expressed as median and interquartile range [M (P25, P75)]. Comparisons among multiple groups were conducted using the Kruskal–Wallis H test, and pairwise comparisons were conducted using the Dunn test with Bonferroni correction. Count data were reported as number of cases (percentage) [n (%)], with intergroup comparisons performed using the chi-squared (χ²) test. Spearman’s rank correlation analysis was used to assess the relationship between serum miRNA-21 and CA15-3 or VEGF levels in patients with BC, as well as to evaluate associations between these markers and clinicopathological characteristics. Receiver operating characteristic (ROC) curve analysis was applied to assess the diagnostic performance of individual and combined markers for BC, including calculation of the area under the curve (AUC), sensitivity, specificity, and 95% confidence intervals (95% CI). The optimal cut-off value of each indicator was determined by the Youden index method. The value corresponding to the maximum Youden index (sensitivity+ specificity-1) was defined as the optimal threshold. Furthermore, the DeLong test was used for pairwise comparison of the area under the ROC curve among the three biomarkers to analyze the statistical differences in differential diagnostic efficacy. All statistical tests were two-tailed, and a P value < 0.05 was considered statistically significant. A binary logistic regression analysis was used to construct a combined diagnostic model incorporating serum miRNA-21, CA15-3, and VEGF. All statistical tests were two-sided, and *p*<0.05 was considered statistically significant.

## 3 Results

### 3.1 Baseline characteristics

A total of 106 patients with early-stage breast cancer were enrolled in this study, and all cases with distant metastasis were excluded. The main clinicopathological features were distributed as follows: Tumor location: left side in 52 cases (49.06%) and right side in 54 cases (50.94%); Histological type: invasive ductal carcinoma was the predominant subtype, accounting for 98 cases (92.45%); the remaining cases included invasive lobular carcinoma in 4 cases (3.77%), invasive papillary carcinoma in 2 cases (1.89%), and other types in 2 cases (1.89%); TNM stage: pT1N0M0 in 63 cases (59.43%), pT2N0M0 in 27 cases (25.47%), pT1N1M0 in 8 cases (7.55%), and pT2N1M0 in 8 cases (7.55%); Clinical stage: stage I in 65 cases (61.32%) and stage II in 41 cases (38.68%), with no stage III/IV cases. Details are shown in **Table 2**.

**Table 2:** Baseline data of the breast cancer group.

Index	Number(n)	Constituent ratio(%)
Age		
≤40	12	11.32
41~50	28	26.42
51~60	35	33.02
61~70	21	19.81
>70	10	9.43
Tumor side		
center	52	49.06
Right	54	50.94
Pathological type		
Invasive ductal carcinoma	98	92.45
Invasive lobular carcinoma	4	3.77
Invasive papillary carcinoma	2	1.89
Other	2	1.89
TNM stages		
pT1N0M0	63	59.43
pT2N0M0	27	25.47
pT1N1M0	8	7.55
pT2N1M0	8	7.55
Clinical stages		
I Stage	65	61.32
II Stage	41	38.68

### 3.2 Comparison of the test results of each indicator among the study participants in the three groups

**Figure 1:**
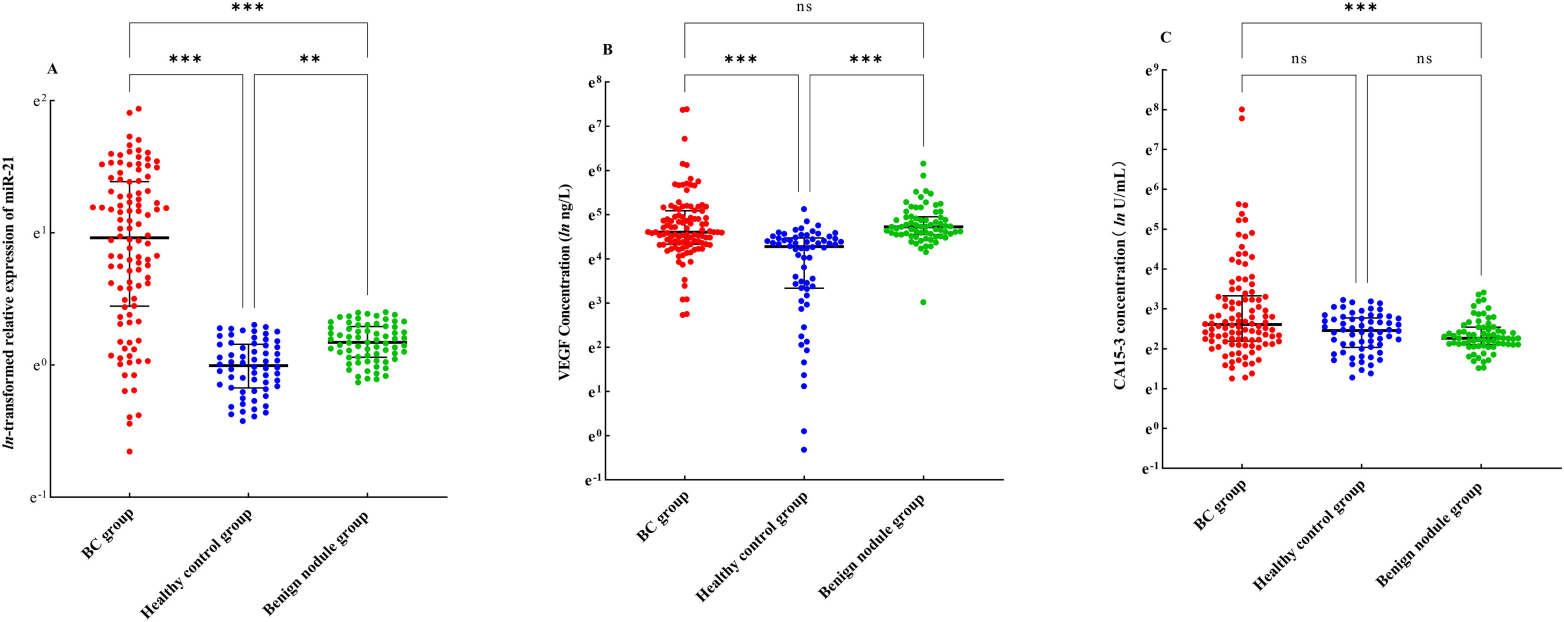
Differential analysis of expression levels of miR-21, VEGF, and CA15-3 among study groups. **(A)** The vertical axis represents the natural log-transformed relative expression level of miR-21 (2^-△△Ct^); red dots = BC group (n = 106), blue dots = healthy control group (n = 62), green dots = benign nodule group (n = 69); horizontal line represents the median, box elements represent the interquartile range (P25-P75). **(B)** The vertical axis represents the natural log-transformed VEGF concentration (ng/L); Grouping, graphical elements, and statistical annotations are the same as in **(A)**. **(C)** The vertical axis represents the natural log-transformed CA15-3 concentration (U/mL); Grouping, graphical elements, and statistical annotations are the same as in **(A)**. Statistical analysis was performed using Kruskal-Wallis test followed by Dunn’s multiple comparisons. ns, p>0.05; ** p<0.01, *** p<0.001.

Statistically significant differences were observed in the overall distribution of miR-21 expression among the three groups: BC, healthy control, and benign nodule (H=105.603, *p*<0.001). The median miR-21 expression level in the BC group was 2.62 (interquartile range: 1.56–4.01), which was significantly higher than that in the healthy control group [1.00 (0.84–1.17)] and the benign nodule group [1.19 (1.06–1.34)] (both *p*<0.05). No significant difference in miR-21 expression was found between the healthy control and benign nodule groups (*p* > 0.05). The distribution of VEGF concentrations differed significantly among the three groups (H=57.326, *p*=0.001). The VEGF concentration in the BC group [100.01 ng/L (76.27–162.06 ng/L)] was significantly higher than that in the healthy control group [72.16 ng/L (28.08–87.74 ng/L)] (*p*<0.05). Additionally, the VEGF concentration in the benign nodule group [112.95 ng/L (94.54–141.37 ng/L)] was significantly higher than that in the healthy control group (*p*<0.05). However, no significant difference in VEGF concentration was observed between the BC and benign nodule groups (*p* > 0.05). Significant differences were identified in CA15-3 concentrations among the three groups (H=14.964, *p*<0.001). The CA15-3 level in the BC group [13.55 U/mL (8.93–27.89 U/mL)] was significantly higher than that in both the healthy control group [11.69 U/mL (7.65–16.01 U/mL)] and the benign nodule group [9.64 U/mL (8.20–12.68 U/mL)] (both *p*<0.05). No significant difference was found between the healthy control and benign nodule groups with respect to CA15-3 levels (*p* > 0.05). Detailed results are presented in **Table 3** and **Figure 1**.

**Table 3:** Comparison of the test results of each indicator among study participants in the three groups [M (P25, P75)].

Groups	miR-21	VEGF(ng/L)	CA15-3(U/mL)
BC group (*n*=106)	2.62(1.56,4.01)^a^	100.01(76.27,162.06)^a^	13.55(8.93,27.89)^b^
Healthy control group (*n*=62)	1.00(0.84,1.17)^b^	72.16(28.08,87.74)	11.69(7.65,16.01)
Benign nodule group (*n*=69)	1.19(1.06,1.34)^ab^	112.95(94.54,141.37)^a^	9.64(8.20,12.68)^b^
H value	105.603	57.326	14.964
*p* value	<0.001	0.001	<0.001

^a^Compared with the control group, *p* <0.05; b. Compared with the benign nodule group, *p* <0.05.

### 3.3 ROC curve analysis of each indicator for the diagnosis of BC

ROC curves were plotted to assess the diagnostic performance of each indicator for BC. The BC group was used as the case group, with the healthy control group and benign nodule group used separately as control groups. For miR-21, the comparison between the BC group and the healthy control group yielded an AUC of 0.897 (95% CI: 0.848–0.946), indicating high diagnostic efficacy. At the optimal cut-off value of 1.36, sensitivity was 81.10% and specificity was 98.40%. In the comparison between the BC group and benign nodule group, the AUC was 0.842 (95% CI: 0.779–0.904), also demonstrating good diagnostic performance. At the optimal cut-off value of 1.52, sensitivity was 76.40% and specificity was 98.60%, indicating that miR-21 maintained high specificity even in the presence of benign breast lesions. For VEGF, the comparison between the BC group and the healthy control group produced an AUC of 0.777 (95% CI: 0.708–0.846), indicating moderate diagnostic performance. At the optimal cut-off value of 91.41 ng/L, the sensitivity and specificity were 61.30% and 80.60%, respectively.

**Figure 2:**
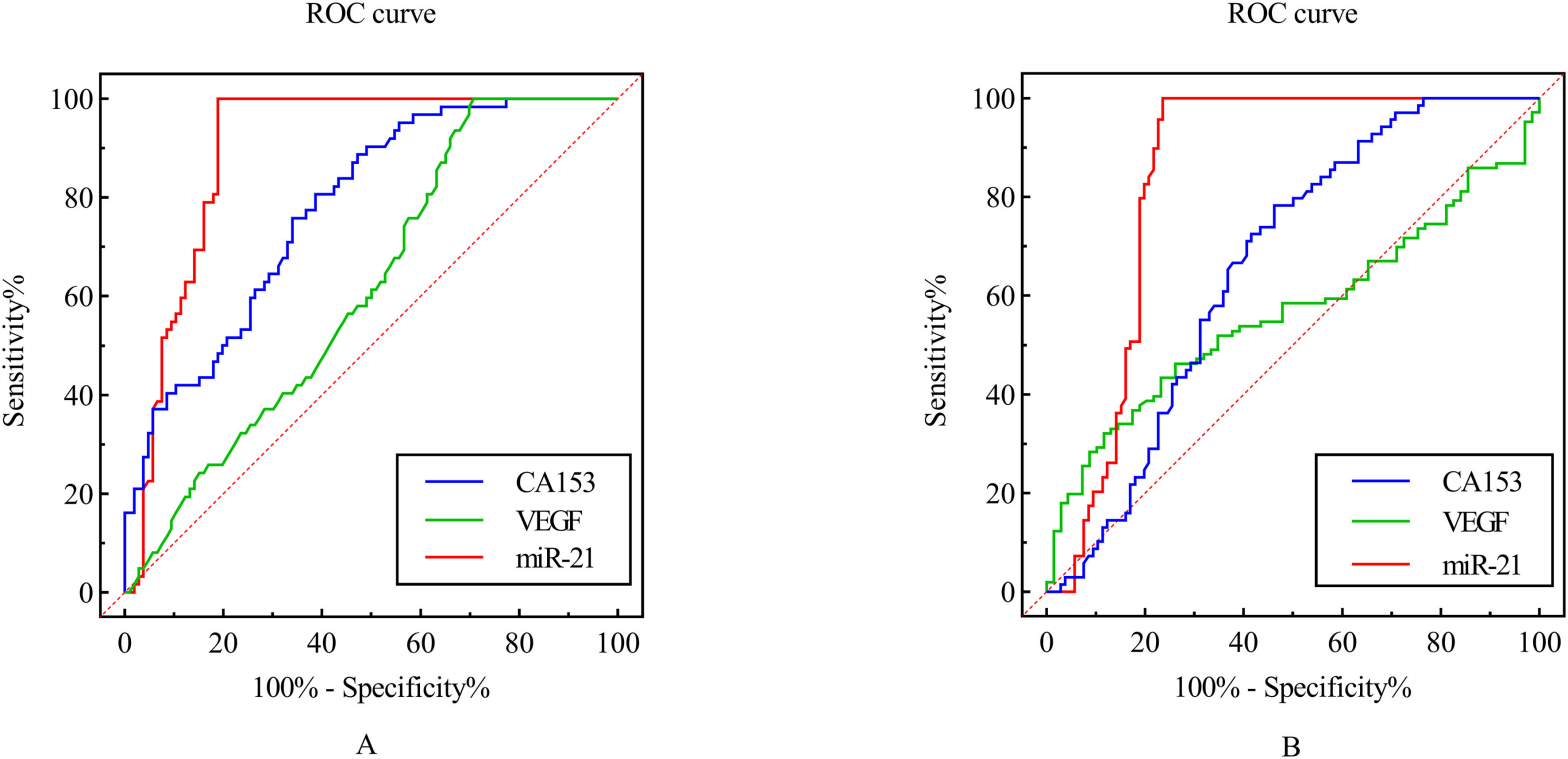
ROC curves of each indicator for the diagnosis of BC. ROC curves represent: miR-21 (red), VEGF (green), and CA15-3 (blue). The dashed line indicates the reference line (AUC = 0.5). Vertical axis: Sensitivity (%); Horizontal axis: 100% – Specificity (%). **(A)** ROC curve for breast cancer group vs. healthy control group **(B)** ROC curve for BC group vs. benign nodule group.

However, in the comparison between the BC group and the benign nodule group, the AUC was 0.433 (95% CI: 0.349–0.518), approaching the reference line (AUC=0.5), with a sensitivity of only 13.20%, indicating that VEGF lacked discriminative power in distinguishing BC from benign nodules. For CA15-3, the AUC comparing the BC group to the healthy control group was 0.615 (95% CI: 0.531–0.699), indicating low diagnostic performance. At the optimal cut-off value of 25.20 U/mL, the sensitivity was 29.20% (indicating a high missed diagnosis rate), although the specificity remained high at 98.40%, suggesting limited clinical value. When compared with the benign nodule group, the AUC was 0.663 (95% CI: 0.583–0.742), demonstrating slightly better performance than in the comparison with healthy controls; however, the overall diagnostic value remained suboptimal, indicating that CA15-3 alone is not suitable as an effective diagnostic marker for BC. Pairwise comparisons of the AUC values among the three biomarkers were performed using the DeLong test. The results showed that the AUC of miR-21 was significantly higher than that of VEGF (Z=7.897, P < 0.001) and CA15-3 (Z=3.449, P=0.001). The AUC of CA15-3 was also significantly higher than that of VEGF (Z =-3.822, P < 0.001). It is suggested that miR-21 exhibits the optimal diagnostic efficacy in differentiating breast cancer from benign breast nodules. Detailed diagnostic parameters are presented in **Tables 4** and **5**, and the corresponding ROC curves are presented in **Figure 2**.

**Table 4:** ROC curve analysis results of each indicator for the diagnosis of BC.

Indicator	Comparison grouping	AUC (95%CI)	Optimal cut-off value	Sensitivity (%)	Specificity (%)
miR-21	BC group vs. Healthy control group	0.897 (0.848~0.946)	1.36	81.10	98.40
	BC group vs Benign nodule group	0.842 (0.779,0.904)	1.52	76.40	98.60
VEGF	BC group vs. Healthy control group	0.777 (0.708,0.846)	91.41ng/L	61.30	80.60
	BC group vs Benign nodule group	0.433 (0.349,0.518)	256.96ng/L	13.20	97.10
CA15-3	BC group vs. Healthy control group	0.615 (0.531,0.699)	25.20U/mL	29.20	98.40
	BC group vs Benign nodule group	0.663 (0.583,0.742)	U/mL	53.80	78.30

**Table 5:** DeLong test for pairwise AUC comparison of biomarkers in the differentiation of breast cancer and benign nodules.

Group	AUC difference	Z value	P value	95%CI (difference)
miR-21 vs VEGF	0.408	7.897	<0.001	0.307~0.509
miR-21 vs CA153	0.179	3.449	0.001	0.077~0.281
VEGF vs CA153	-0.229	-3.822	<0.001	-0.347~0.347

### 3.4 Correlation analysis between miR-21 and raditional serum biomarkers

Spearman’s non-parametric correlation analysis was conducted to assess the association between serum miR-21 expression and VEGF and CA15-3 levels in patients with BC. As presented in **Table 6**, There was a statistically significant weak positive correlation between miR-21 and VEGF *r*=0.239*,p*<0.001, suggesting that both markers may share an upward expression trend in breast cancer. However, given the low correlation coefficient, a direct regulatory relationship between miR-21 and VEGF remains to be further verified. No significant correlation was identified between the relative expression of serum miR-21 and CA15-3 levels (r = 0.103, *p*=0.113).

**Table 6:** Correlation analysis between serum miR-21 and VEGF, CA15-3 in patients with BC.

Paired Indicators	Correlation coefficient (*r*)	*P* value (two-tailed)
miR-21 vs VEGF	0.239	<0.001
miR-21 vs CA15-3	0.103	0.113
VEGF vs CA15-3	0.035	0.587

## 4 Discussion

Breast cancer is a highly prevalent malignant tumor affecting women globally. Its heterogeneous biological characteristics and insidious onset severely hinder early clinical detection, resulting in missed optimal intervention timing and poor long-term survival for numerous patients (12). Although imaging screening is widely utilized for routine screening, it exhibits insufficient sensitivity in individuals with dense breast tissue. Meanwhile, conventional serum biomarkers generally yield suboptimal diagnostic efficacy in the early stage of tumor progression and cannot satisfy the clinical requirements for accurate disease stratification (13). Therefore, identification of novel liquid biopsy biomarkers with favorable sensitivity and specificity to overcome current diagnostic limitations and optimize prognostic management has emerged as a research hotspot and urgent clinical demand in oncology. Among the biomarkers commonly used in clinical cancer diagnostics, extracellular non-coding RNAs have emerged as a prominent focus in molecular diagnostics, particularly as RNA-based biomarkers. Within this category, miRNAs have garnered considerable attention due to their extensive diversity and notable stability in body fluids (14). miR-21, located on chromosome 17q21.3, is among the most frequently upregulated miRNAs in cancer and silences multiple target genes implicated in oncogenic signaling pathways (15). Mounting evidence has demonstrated that miR-21 is significantly overexpressed in breast cancer tissues. It modulates target genes and signaling pathways in cancer stem cells to regulate cell proliferation, apoptosis, invasion, metastasis, self-renewal and growth, thereby affecting disease progression and clinical prognosis of breast cancer (16–21). Given the importance of early detection in improving prognosis for patients with BC, the identification of biomarkers with high sensitivity and specificity remains a central focus of clinical research. In this context, Adopting a rigorous case-control design, this study enrolled three cohorts, including patients with breast cancer, individuals with benign breast nodules, and healthy controls, at a single center to systematically compare the expression profiles and diagnostic values of circulating miR-21, VEGF and CA15-3. With the assistance of receiver operating characteristic curve analysis and the DeLong test, we quantitatively evaluated the discriminatory capacity of each biomarker and further highlighted the outstanding performance of miR-21 in differentiating malignant breast lesions from benign conditions. First of all, the results demonstrated that miR-21 yielded a distinctly higher area under the curve and superior specificity compared with conventional indicators, suggesting its great potential as an independent diagnostic biomarker for breast cancer. Next in importance, the findings of this study indicated that the relative expression level of serum circulating miR-21 in the BC group was significantly elevated compared with that in both the healthy control group and the benign nodule group, indicating upregulated miR-21 expression is closely linked to BC occurrence, which confers it prominent diagnostic efficacy. These results are consistent with previously reported findings (20–25).

The molecular basis of this phenomenon lies in the systemic inhibition of the apoptosis pathway by miR-21 as a key oncogene. Current evidence indicates that miR-21 directly targets pro-apoptotic genes such as Fas ligand (FasL), programmed cell death 4 (PDCD4) and phosphatase and tensin homolog (PTEN), thereby inhibiting cancer cell apoptosis and promoting sustained proliferation and invasion (12). This post-transcriptional modulation mechanism explains the upregulation of miR-21 in tumor tissues, and also clarifies its biological characteristics: miR-21 can be packaged into exosomes and secreted into the peripheral circulation with stable expression, making it a credible liquid biopsy indicator for reflecting tumor burden (26). Although previous studies have suggested heterogeneous associations of miR-21 expression with breast cancer molecular subtypes and lymph node metastasis (27). The consistently elevated miR-21 expression observed in our cohort aids in distinguishing benign from malignant breast lesions, further supporting its promising potential as a practical auxiliary biomarker for breast cancer differential diagnosis (28). It may help mitigate the false-positive defects of conventional imaging examinations in clinical practice. Collectively, miR-21-mediated activation of anti-apoptotic signaling pathways serves as the core molecular mechanism underlying its favorable diagnostic performance in peripheral blood testing. In contrast, VEGF concentrations were significantly elevated in both the BC and benign nodule groups compared with the healthy control group; however, no significant difference was observed between the BC and benign nodule groups. This pattern suggests that elevated VEGF expression may reflect abnormal hyperplastic changes in breast tissue in general, rather than representing a BC–specific alteration. This observation is consistent with the low diagnostic accuracy of VEGF in differentiating BC from benign nodules (AUC=0.433).

CA15-3, a conventional tumor marker associated with BC, was significantly increased in the BC group in this study. However, its sensitivity was only 29.20%, resulting in a high rate of missed diagnoses. This finding aligns with the established clinical understanding that CA15-3 is more appropriate for monitoring therapeutic response rather than for early diagnostic screening. Correlation analysis conducted in this study indicated no statistically significant relationship between serum miR-21 and either VEGF or CA15-3 levels in patients with BC. This finding indicates that miR-21 may be involved in the development and progression of BC through pathways independent of angiogenesis and raditional serum biomarkers.

The regulatory mechanisms underlying miR-21 expression may be more complex and warrant further investigation. ROC curve analysis is a widely accepted method for assessing the diagnostic performance of clinical indicators. In this study, miR-21 demonstrated high diagnostic efficacy in distinguishing the BC group from the healthy control group, with an AUC of 0.897, sensitivity of 81.10%, and specificity of 98.40%. When differentiating the BC group from the benign nodule group, the AUC remained high at 0.842, and the specificity reached 98.60%. These findings indicate that miR-21 retains strong discriminatory power even in benign breast lesions, which are frequently encountered as clinical confounders. This characteristic has important implications for diagnostic accuracy and may help serve as an adjunctive marker to improve diagnostic specificity, which could indirectly help reduce unnecessary follow-up procedures in future clinical applications. In contrast, VEGF demonstrated only moderate diagnostic performance in distinguishing BC from healthy individuals, while CA15-3 indicated limited diagnostic value overall.

These results indicate that neither VEGF nor CA15-3 is appropriate for use as a standalone biomarker in the early detection of BC. This study has several limitations. The sample size was relatively small, and the investigation was conducted at a single center, which may introduce selection bias. In addition, the relationship between miR-21 expression and clinicopathological features of BC such as tumor stage, histological grade, and lymph node metastasis was not examined. Furthermore, although we evaluated individual biomarker performance, we did not conduct combined detection or analysis of multiple markers in this cohort. Thus, the potential added value of a multi-marker panel for improving diagnostic performance remains to be validated in future studies.

## 5 Conclusion

In summary, the findings of this study confirm that serum miR-21 is significantly overexpressed in patients with BC. Its diagnostic performance distinguishing BC from both healthy individuals and those with benign breast nodules was superior to that of VEGF and the traditional tumor marker CA15-3. Pearson correlation analysis revealed a significant weak positive correlation between serum miR-21 and VEGF in BC patients, while no significant correlation was observed between miR-21 and CA15-3, indicating that miR-21 may contribute to BC pathogenesis through an independent molecular pathway. These findings support the potential of miR-21 as a promising adjunctive biomarker for breast cancer differential diagnosis. Further multicenter, large-scale studies with stage-stratified cohorts are required to validate its clinical utility and explore its biological roles in breast cancer progression. However, certain limitations must be acknowledged. These include a relatively small sample size, the single-center design, and the lack of investigation into the association between miR-21 expression and clinicopathological characteristics, as well as the absence of combined analysis involving multiple biomarkers. Future studies should incorporate multicenter designs with larger sample sizes to further elucidate the biological mechanisms underlying miR-21 expression and to validate its combined diagnostic use alongside other markers, thereby providing more robust clinical evidence for candidate biomarker for breast cancer diagnosis and differential diagnosis.

## Data Availability

The original contributions presented in the study are included in the article/supplementary material. Further inquiries can be directed to the corresponding author.
